# "Predictability of body mass index for diabetes: Affected by the presence of metabolic syndrome?"

**DOI:** 10.1186/1471-2458-11-383

**Published:** 2011-05-25

**Authors:** Farzad Hadaegh, Mohammadreza Bozorgmanesh, Maryam Safarkhani, Davood Khalili, Fereidoun Azizi

**Affiliations:** 1 Prevention of Metabolic Disorders Research Center, Research Institute for Endocrine Sciences, Shahid Beheshti University of Medical Sciences, Tehran, IRAN; 2 Endocrine Research Center, Research Institute for Endocrine Sciences, Shahid Beheshti University of Medical Sciences, Tehran, IRAN

**Keywords:** diabetes, prediction, metabolic syndrome, body mass index

## Abstract

**Background:**

Metabolic syndrome (MetS) and body mass index (BMI, kg.m^-2^) are established independent risk factors in the development of diabetes; we prospectively examined their relative contributions and joint relationship with incident diabetes in a Middle Eastern cohort.

**Method:**

participants of the ongoing Tehran lipid and glucose study are followed on a triennial basis. Among non-diabetic participants aged≥ 20 years at baseline (8,121) those with at least one follow-up examination (5,250) were included for the current study. Multivariate logistic regression models were used to estimate sex-specific adjusted odd ratios (ORs) and 95% confidence intervals (CIs) of baseline BMI-MetS categories (normal weight without MetS as reference group) for incident diabetes among 2186 men and 3064 women, aged ≥ 20 years, free of diabetes at baseline.

**Result:**

During follow up (median 6.5 years); there were 369 incident diabetes (147 in men). In women without MetS, the multivariate adjusted ORs (95% CIs) for overweight (BMI 25-30 kg/m2) and obese (BMI≥30) participants were 2.3 (1.2-4.3) and 2.2 (1.0-4.7), respectively. The corresponding ORs for men without MetS were 1.6 (0.9-2.9) and 3.6 (1.5-8.4) respectively. As compared to the normal-weight/without MetS, normal-weight women and men with MetS, had a multivariate-adjusted ORs for incident diabetes of 8.8 (3.7-21.2) and 3.1 (1.3-7.0), respectively. The corresponding ORs for overweight and obese women with MetS reached to 7.7 (4.0-14.9) and 12.6 (6.9-23.2) and for men reached to 3.4(2.0-5.8) and 5.7(3.9-9.9), respectively.

**Conclusion:**

This study highlights the importance of screening for MetS in normal weight individuals. Obesity increases diabetes risk in the absence of MetS, underscores the need for more stringent criteria to define healthy metabolic state among obese individuals. Weight reduction measures, thus, should be encouraged in conjunction with achieving metabolic targets not addressed by current definition of MetS, both in every day encounter and public health setting.

## Background

Diabetes is "a common, growing, serious, costly, and potentially preventable public health problem" [1]. Metabolic syndrome (MetS) and body mass index (BMI) are established independent risk factors in the development of diabetes [2]. Obesity consists of heterogeneous phenotypes resulting from interplay between genetic and environmental factors [3]. Increased BMI has been associated with metabolic and cardiovascular risk factors including diabetes, hypertension, dyslipidemia, but there is increasing evidence that sub-phenotypes of obesity exist that appear to deviate from the standard dose-response relationship between increased BMI and its adverse clinical outcomes [4]. Metabolically obese but normal-weight (normal-weight/MetS) is a condition to be ascertained in individuals who despite having a normal-weight BMI, present metabolic disturbances typical of obese individuals. This constellation, generally known as MetS, includes impaired insulin sensitivity, increased visceral adiposity, low levels of high-density lipoprotein cholesterol (HDL-C), elevated levels of fasting glucose and triglycerides (TGs), and hypertension [5-9]. It has been shown that the normal-weight/MetS phenotype is associated with a three- to fourfold higher risk for diabetes as compared with control subjects [10]. On the other hand, metabolically healthy but obese (obese/without MetS) individuals, have been identified who, despite having BMI exceeding 30 kg.m^-2^, are relatively insulin sensitive and have a rather favorable cardiovascular risk profile [11-14] with a three- to fourfold lower risk for diabetes as compared with obese insulin-resistant individuals [10]. There has been, however, no consensus regarding the definitions of obese/without MetS [15-18] and the existence of a healthy obese phenotype based on the definition of absence of MetS [19] has recently been questioned [20]. Since no population-based study prospectively has examined sex-specifically the joint relationship between BMI and MetS with diabetes [10], an unanswered question remains to be whether the impact of diagnosis of different obesity phenotype on prediction of incident diabetes differs by sex.

Plethora of evidences currently supports the notion that diabetes can be prevented or the onset delayed [21,22]. In this light, the need for evidence-based guidelines for putting prevention into practice is seen as a public health priority. It is, therefore, worthwhile to clarify the combined effect of BMI and MetS for public health systems in order to best support people by stratifying them by risk and in turn target those at highest risk of developing diabetes in the future. Therefore, using data based on a Middle Eastern population, we investigated the combined relationship of BMI and MetS with incident diabetes to understand if MetS modify increasing effect of BMI on diabetes.

## Methods

### Study population

**Figure 1:**
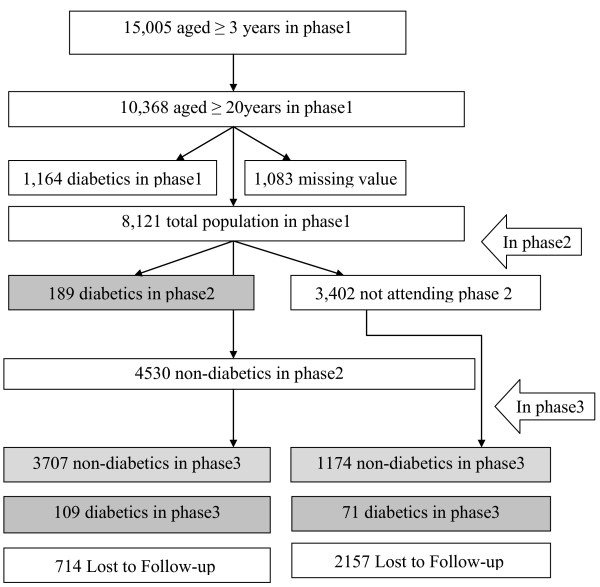
**Study population, inclusions and exclusions**.

The Tehran Lipid and Glucose Study (TLGS) is a prospective population based study performed on a representative sample of the Tehran population, with the aim of determining the prevalence of non-communicable disease (NCD) risk factors and developing a healthy lifestyle to improve them [11,12]. The baseline survey was performed from February 1999 to July 2001(phase 1) and 4751 families, which included more than 15,000 residents of district-13 of Tehran aged ≥3 years were selected by cluster random-sampling method. After this cross-sectional phase, subjects entered into a cohort and a prospective interventional study (lifestyle modification education). The current study used the data from 10,368 individuals ≥ 20 years who had baseline examination. After exclusion of participants with prevalent diabetes (n = 1164), and those with missing data regarding fasting and 2 hour post challenge plasma glucose (2 h-PCPG) (n = 884), BMI and MetS definition (n = 199), 8,121 non-diabetic participants remained eligible to be reexamined in two consecutive phases, one from September 2001 to August 2005 (phase 2) and the other from April 2005 to March 2008 (phase3). The same standard approach is followed to collect information across consecutive examinations of the TLGS follow up study. Participants with at least one follow-up examination (5,250) were included for the current study (Figure 1).

Informed written consent was obtained from all participants and the ethical committee of the Research Institute for Endocrine Sciences approved this study.

### Clinical and laboratory measurements

A trained interviewer collected information using a pretested questionnaire. The information obtained included demographic data, family history of diabetes and past medical history of cardiovascular disorder (CVD), drug use and smoking behavior. Weight was measured, with subjects minimally clothed without shoes, using digital scales (Seca 707: range 0.1-150 kg) and recorded to the nearest 100 g. Height was measured in a standing position without shoes, using tape meter while shoulders were in a normal alignment. Waist circumference (WC) was measured at the umbilical level. Two measurements of systolic blood pressure (SBP) and diastolic blood pressure (DBP) were taken using a standardized mercury sphygmomanometer on the right arm, after a 15 minute rest in a sitting position; mean of the two measurements was considered as the subject's blood pressure [12].

Fasting plasma glucose (FPG), serum HDL-C, and TGs levels were measured by previously reported methods [23,24]. BMI was calculated as weight (kg) divided by square of the height (m^2^).

### Definition of terms

The normal-weight/MetS phenotype was defined as the status of having normal weight (BMI < 25 kg.m^-2^) but meeting the MetS criteria. In contrast, obese/without MetS phenotype was defined as the status of being obese or overweight (BMI ≥ 25 kg.m^-2^), but not having MetS [18]. We used the updated harmonized definition of MetS [25,26]. We used WC cutoff points known to be appropriate for Persian men and women [27]. MetS was ascertained in individuals meeting three or more of the following criteria. (1) Waist circumference ≥ 94.5 cm. (2) HDL-C <1.04 mmol.l^-1 ^(40 mg.dl^-1^) in men and <1.30 mmol.l^-1 ^(50 mg.dl^-1^) in women. (3) TGs ≥1.7 mmol.l^-1 ^(150 mg.dl^-1^) or specific treatment for this lipid abnormality. (4) Hypertension defined as SBP ≥130 mmHg or DBP ≥85 mmHg or treatment of previously diagnosed hypertension. (5) fasting glucose ≥5.5 mmol.l^-1 ^(100 mg.dl^-1^) or previously diagnosed diabetes [28]. Smoking status included a record of current, occasional and past smoking history (those who had never smoked were called non-smokers). Positive family history of diabetes was defined as having at least one parent or sibling with diabetes. Participants were classified as having diabetes at the baseline or during follow-up if they met at least one of these criteria: FPG ≥7 mmol.l^-1^, or 2 h-PCPG≥11.1 mmol.l^-1 ^or taking anti-diabetic medication [29]. A previous history of CVD reflected any prior diagnosis of CVD by a physician.

### Statistics

To investigate the sex-specific combined effect of BMI and MetS on the development of diabetes, joint BMI-MetS variable was created. That is, participants were divided into groups based on both their BMI (kg.m^-2^) (normal weight, <25, overweight 25-30, and obese ≥ 30) [30] and MetS status [26]. Mean (SDs) were calculated for all continuous variables and the percentage of participants in each BMI-MetS category was determined for all categorical variables. Values for TGs levels were log-transformed since the distribution was highly skewed. Differences among BMI-MetS categories were examined by Chi-square or ANOVA where appropriate. The cumulative incidence of diabetes was calculated as the number of diabetes events divided by the number of subjects at risk in each category of BMI-MetS. The independent and combined effects on diabetes of both BMI and MetS were examined using multivariable logistic regression models separately for each sex. We chose our candidate covariates among the ones that were validated from the literature and new ones that are suspected of playing important roles in the development of diabetes [31,32]. As such, our covariate selection can be regarded as being guided by scientific as well as numeric evidence. The following variables served as standard candidate risk factors: age, sex, body mass index (BMI), waist circumference, exercise, diabetes, SBP, DBP, smoking, family history of diabetes, TGs, and HDL-C [31]. We followed statistical guidelines with respect to the significance of association of a variable with incident diabetes but also considered scientific and qualitative judgment as well. For example we did not adjusted for waist circumference, TGs, HDL-C, and FPG which are components of the MetS and therefore not appropriate to be adjusted for in prediction models already incorporating MetS. Sex-specific odds ratios (ORs) and their 95% confidence intervals (CIs) were calculated using age- and multivariate-adjusted models, which included age, family history of diabetes, history of cardiovascular disease (CVD), education, smoking and the TLGS interventions. Age- and multivariate-adjusted logistic regression models incorporating the joint variable were also created. The normal weight individuals, without MetS were the referent group when the combined effect of the MetS and BMI were to be evaluated. When BMI was the predictor under evaluation, the reference group was normal weight individuals, regardless of MetS status. When MetS was the predictor under evaluation, the participants without MetS were considered as reference group. Hosmer-Lemeshow test for goodness-of-fit was implemented to assess the calibration of logistic models [33]. Linearity in the regression coefficients of the continuous covariates were analyzed by using multivariate restricted cubic splines [34].

Alternate analyses using Cox proportional hazards models that accounted for interval censoring gave the same results hence, only logistic regression results are presented. Wald tests of the linear hypotheses concerning the logistic regression models coefficients were performed to test the null hypotheses that the ORs (effect size) for one MetS-BMI category was equal to that of another category. All statistical analyses were performed using STATA version 10.0 (StataCorp LP, College Station, Texas) and values of P < 0.05 from 2-sided tests were considered statistically significant.

## Results

Non-diabetic participants who did not attend the follow up study have been considered as non-participants. At baseline, as compared to non-participants, participants had higher BMI (26.8 vs. 26.4 kg.m-2), WC (88 vs. 87 cm), TG levels (1.59 vs. 1.51 mmol.l^-1^), and prevalence of family history of diabetes (26% vs. 24%). The prevalence of smoking (19% vs. 25%) and CVD (3.2% vs. 5.1%) and the proportion of participants assigned to the intervention measures (36% vs. 41%) were lower among participants. No significant difference was found between the two groups with respect to age, systolic and diastolic blood pressure, FPG, 2 h-PCPG and HDL-C.

The baseline characteristics of the participants by BMI-MetS categories are presented in Table 1 and 2. In both sexes, all components of MetS as well as age, history of CVD and family history of diabetes and educational status varied significantly by joint BMI-MetS status. Among 2,189 men, 4.3% were normal weight with MetS (resembling the normal-weight/MetS phenotype), and 29% were overweight or obese without MetS (resembling the obese/without MetS phenotype). About 38.1% of the participants were normal weight, without MetS, and 28.8% were overweight or obese with MetS. Among normal-weight participants, 10.1% had MetS, and among overweight and obese subjects, 50.1% did not have MetS. Among women (n = 3,064), 2.3% represented the normal-weight/MetS phenotype, and 41.7%, the obese/without MetS phenotype; 28.0% of women were normal weight, without MetS, and 27.9% were overweight or obese with MetS. Among normal-weight women, 7.7% had MetS, and among overweight and obese subjects, 59.9% did not have MetS.

**Table 1:** Distribution of baseline characteristics of the women across BMI-MetS categories among women

Women	Normal weight	Normal weight	Overweight	Overweight	Obese	Obese	
	Without MetS	With MetS	Without MetS	With MetS	Without MetS	With MetS	p
	(N = 859)	(N = 72)	(N = 907)	(N = 331)	(N = 371)	(N = 524)	
Diabetic, n (%)	14(1.6)	11(15.3)	35(3.9)	42(12.7)	14(3.8)	106(20.2)	
Age, y	33.4(10.63)	52.3(11.79^a^	39.2(11.20)^a^	49.4(11.92)^a^	41.2(10.98)^a^	48.2(10.84)^a^	<0.001
BMI, kg.m-2	22.1(2.12)	23.4(1.33)^a^	27.3(1.41)^a^	27.8(1.38)^a^	32.7 (2.69)^a^	33.8(3.24)^a^	<0.001
Waist, cm	74.9(7.63)	82.1(8.08)^a^	85.3(6.92)^a^	92.4(7.45)^a^	95.0 (8.68)^a^	102.5(7.90)^a^	<0.001
SBP, mmHg	107.6(11.70)	131.2(17.44)^a^	113.0(13.68)^a^	130.9(19.74)^a^	116.6 (14.42)^a^	129.3(19.64)^a^	<0.001
DBP, mmHg	71.7(8.36)	82.5(8.34)^a^	75.1(8.20)^a^	84.5(9.55)^a^	78.0 (9.06)^a^	84.7(10.14)^a^	<0.001
FPG, mmol/L	4.75(0.44)	5.30(0.56)^a^	4.87(0.45)^a^	5.23(0.59)^a^	4.82 (0.45)	5.27(0.59)^a^	<0.001
2hPCPG, mmol/L	5.42(1.26)	6.77(1.58)^a^	5.90(1.39)^a^	6.92(1.64)^a^	5.8 9(1.33)^a^	7.00(1.61)^a^	<0.001
HDL-C, mmol/L	1.24(0.29)	1.09(0.20)^a^	1.18(0.29)^a^	1.04(0.21)^a^	1.24 (0.30)	1.05(0.23)^a^	<0.001
Triglycerides, mmol/L	1.04(1.01-1.07)	2.31 (2.12-2.51)^a^	1.36 (1.32-1.4)^a^	2.35(2.25-2.46)^a^	1.34 (1.29-1.4)^a^	2.32 (2.24-2.41)^a^	<0.001
Intervention, (%)	301(35.0)	29(40.3)	337(37.2)	123(37.2)	131(35.3)	187(35.7)	0.890
Smoking (%)	29(3.4)	3(4.2)	51(5.6)	15(4.5)	17 (4.6)	30(5.7)	0.273
Hypertension, (%)	36(4.2)	35(48.6)^†^	66(7.3)^†^	159(48.3)^b^	53 (14.3)^b^	243(46.6)^b^	<0.001
History of CVD, (%)	4(0.5)	4(5.6)^b^	18(2.0)^b^	18(5.4)^b^	8 (2.2)^b^	26(5.0)^b^	<0.001
Education, (%) Illiterate	139(16.2)	48(66.7)^b^	256(28.2)^b^	189(57.1)^b^	138 (37.2)^b^	327(62.4)^b^	<0.001
Under diploma	589(68.6)	19(26.4)^b^	559(61.6)^b^	131(39.6)^b^	209 (56.3)^b^	183(34.9)^b^	
Over diploma	131(15.3)	5(6.9)	92(10.1)^b^	11(3.3)^b^	24 (6.5)^b^	14(2.7)^b^	
Family history DM,(%)	196(22.8)	20(27.8)	261(28.8)^b^	91(27.5)	105 (28.3)	170(32.4)^b^	0.005

BMI, body mass index; CVD: Cardiovascular disease, DBP, diastolic blood pressure; FPG, fasting plasma glucose, HDL-C: High density lipoprotein cholesterol, MetS, metabolic syndrome; SBP, systolic blood pressure; 2hPCPG; 2 hours post-challenge plasma glucose,

Data has been represented either as mean (SD) for continuous variables or n(%) for categorically distributed variables ANOVA; % is shown for categorical variables with P value according to chi-square; TG was log-transformed and is shown as geometric mean (CI).

BMI (kg.m^-2^) categories: normal <25; overweight, 25-30; and obese ≥ 30.

Hypertension: Blood pressure ≥ 140/90 and/or taking of antihypertensive medication.

aP < 0.05 compared with normal-weight participants without MetS. (base on Dunnett t-test).

^b ^P < 0.01 compared with normal-weight participants without MetS. (base on chi-square test).

**Table 2:** Distribution of baseline characteristics of the men across BMI-MetS categories among men.

Men	Normal weight	Normal weight	Overweight	Overweight	Obese	Obese	
	Without MetS	With MetS (N = 93)	Without MetS	With MetS	Without MetS	With MetS	p
	(N = 830)		(N = 552)	(N = 400)	(N = 81)	(N = 230)	
Diabetic, (%)	23(2.8)	9(9.7)	26(4.7)	43(10.8)	8(9.9)	38(16.5)	
Age, y	41.0(14.64)	49.4(15.64)^a^	41.9(12.98)	47.9(13.39)^a^	42.0(12.90)	44.8(13.34)^a^	<0.001
BMI, kg.m-2	22.2(2.00)	23.4(1.39)^a^	26.9(1.31)^a^	27.6(1.38)^a^	31.9(1.84)^a^	32.6(2.96)^a^	<0.001
Waist, cm	78.80(6.66)	84.39(6.49)^a^	90.38(5.32)^a^	95.06(5.77)^a^	101.35(6.90)^a^	105.08(7.87)^a^	<0.001
SBP, mmHg	113.8(14.96)	127.9(16.19)^a^	116.4(13.58)^a^	129.2(18.34)^a^	118.6(16.37)^a^	128.9(17.36)^a^	<0.001
DBP, mmHg	73.1(9.30)	82.3(10.30)^a^	76.5 (8.92)^a^	83.9(10.55)^a^	78.1(8.78)^a^	84.9(10.39)^a^	<0.001
FPG, mmol/L	4.89(0.45)	5.35(0.64)^a^	4.96(0.47)	5.27(0.58)^a^	4.99(0.35)	5.27(0.55)^a^	<0.001
2hPCPG, mmol/L	5.19(1.45)	6.33(1.85)^a^	5.66(1.59)^a^	6.30(1.88)^a^	6.11(1.77)^a^	6.32(1.85)^a^	<0.001
HDL-C, mmol/L	1.06(0.24)	0.85(0.15)^a^	1.00(0.23^a^	0.89(0.18^a^	1.09(0.23)	0.89(0.19)^a^	<0.001
Triglycerides, mmol/L	1.3(1.26-1.35)	2.6 (2.36-2.77)^a^	1.7 (1.61-1.75)^a^	2.6(2.45-2.68)^a^	1.5(1.34-1.68)	2.5 (2.31-2.60)^a^	<0.001
Intervention, n (%)	285(34.3)	21(22.6)	209(37.9)	141(35.3)	33(40.7)	92(40.0)	0.042
Smoking (%)	362(43.9)	37(39.8)	212(38.4)	161(40.5)	22(27.2)^b^	96(41.9)	0.052
Hypertension, n (%)	64(7.7)	36(38.7)^b^	57(10.3)	158(39.7)^b^	9(11.1)	87(38.2)^b^	<0.001
History of CVD, n (%)	20(2.4)	8(8.6)^b^	18(3.3)	30(7.5)^b^	1(1.2)	14(6.1)^b^	<0.001
Education, n (%)							<0.001
Illiterate	167(20.1)	30(32.3)^b^	116(21.0)	126(31.5)^b^	24(29.6)	68(29.6)^b^	
Under diploma	496(59.8)	45(48.4)	329(59.6)	204(51.0)^b^	48(59.3)	126(54.8)	
Over diploma	167(20.1)	18(19.4)	107(19.4)	70(17.5)	9(11.1)	36(15.7)	
Family history DM (%)	176(21.2)	18(19.4)	146(26.4)	105(26.3)	21(25.9)	81(35.2)^b^	0.001

BMI, body mass index; CVD: Cardiovascular disease, DBP, diastolic blood pressure; FPG, fasting plasma glucose, HDL-C: High density lipoprotein cholesterol, MetS, metabolic syndrome; SBP, systolic blood pressure; 2hPCPG; 2 hours post-challenge plasma glucose,

Data has been represented either as mean (SD) for continuous variables or n(%) for categorically distributed variables ANOVA; % is shown for categorical variables with P value according to chi-square; TG was log-transformed and is shown as geometric mean (CI).

BMI (kg.m^-2^) categories: normal <25; overweight, 25-30; and obese ≥ 30.

Hypertension: Blood pressure ≥ 140/90 and/or taking of antihypertensive medication.

aP < 0.05 compared with normal-weight participants without MetS. (base on Dunnett t-test).

^b ^P < 0.01 compared with normal-weight participants without MetS. (base on chi-square test).

During follow-up (median 6.5 years), diabetes was ascertained in 369 of participants (cumulative incidence: men 6.7% and women 7.2%). Cumulative incidences of diabetes among normal weight, overweight, and obese men were 3.5, 7.2 and 14.8%, respectively. The corresponding figures among women were 2.7, 6.2, and 13.4%.

**Figure 2:**
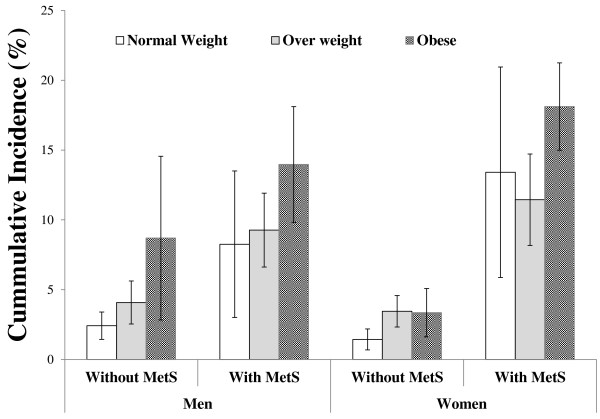
**The cumulative incidence of diabetes across BMI-MetS categories**. Among men (left) and women (right). MetS, metabolic syndrome. Normal weight, <25 (kg.m^-2^); overweight, 25-30 (kg.m^-2^); and obese, ≥30 (kg.m^-2^).

The cumulative incidence of diabetes stratified by BMI-MetS categories in each sex are shown in the Figure 2. The cumulative incidence of diabetes were generally higher among participants with MetS as compared to those without MetS. In men, the cumulative incidence of diabetes increase with increasing levels of BMI, regardless of MetS status. In women, among those without MetS the cumulative incidence of diabetes was relatively the same across BMI groups, whereas among those with MetS the highest incidence was observed in obese women.

The individual effects of BMI and MetS on diabetes in both age- and multivariate-adjusted models are presented in Table 3. As compared to normal-weight men, overweight men had an increased risk of diabetes with an age-adjusted OR of 2.1 (95%CI 1.3-3.0), and obese men had an even greater OR of 4.7 (95%CI 2.6-6.8). Among women, the corresponding figures were 2.0 (95%CI 1.2-3.1) and 4.2 (95%CI 2.6-6.7), respectively. As compared to those without MetS participants who met the MetS criteria had an increased risk of incident diabetes with age-adjusted OR of 3.1 (95%CI 2.2-4.4) and 6.1 (95%CI 4.4-8.5) among men and women, respectively (Table 3). Adjustment for confounders attenuated the association with both MetS and BMI; however, the relationship remained significant among women.

**Table 3:** Contribution of individual and combined effects of BMI and MetS to the risk of incident diabetes.

	Men						Women					
	
	Age adjusted			Multivariable-adjusted^a^			Age adjusted			Multivariable-adjusted^a^		
	
	OR	95% CIs	P	OR	95% CIs	P	OR	95% CIs	P	OR	95% CIs	P
MetS^b^	3.1	2.2-4.4	<0.001	2.9	2.1-4.2	<0.001	6.1	4.4-8.5	<0.001	5.8	4.2-8.2	<0.001
BMI (kg.m^-2^)^c^												
<25	1.0			1.0			1.0			1.0		
25-30	2.1	1.3-3.2	0.001	1.9	1.3-3.0	0.003	2.0	1.2-3.1	0.005	1.9	1.2-3.1	0.006
≥30	4.7	2.93-7.6	<0.001	4.2	2.6-6.8	<0.001	4.2	2.7-6.7	<0.001	3.9	2.5-6.3	<0.001
No MetS-BMI (kg.m^-2^)^d^												
<25	1.0			1.0			1.0			1.0		
25-30	1.7	1.0-3.1	0.063	1.6	0.9-2.9	0.096	2.3	1.2-4.3	0.010	2.3	1.2-4.3	0.012
≥30	3.8	1.7-8.9	0.002	3.6	1.5-8.4	0.003	2.2	1.0-4.7	0.039	2.2	1.0-4.7	0.044
MetS-BMI (kg.m^-2^)												
<25	3.0	1.3-6.9	0.007	3.1	1.3-7.0	0.007	9.3	3.9-22.1	<0.001	8.8	3.7-21.2	<0.001
25-29.9	3.6	2.1-6.1	<0.001	3.4	2.0-5.8	<0.001	7.7	4.0-14.7	<0.001	7.7	4.0-14.9	<0.001
≥30	6.5	3.8-11.2	<0.001	5.7	3.3-9.9	<0.001	13.5	7.4-24.6	<0.001	12.6	6.9-23.2	<0.001

BMI, body a mass index, calculated as weight (kg) divided by height (m) squared; MetS, metabolic syndrome.

a. OR adjusted for age, family history of diabetes, history of CVD, education, smoking, and intervention

b. Reference: individuals without MetS

c. Reference: normal-weight individuals

d. Reference: normal-weight individuals without MetS

Examination of the combined effect of BMI and MetS is shown in Table 3. With the exception of the overweight men without MetS, each group had a statistically significant increased multivariate-adjusted risk of diabetes compared with the normal-weight individuals without MetS. When the individuals with and without MetS within the same BMI strata were compared with Wald test, multivariate-adjusted OR increased from 1.6 to 3.4 (*P *= 0.052) for overweight men and from 2.3 to 7.7 (*P *= 0.001) for overweight women; and from 3.6 to 5.7 (*P *= 0.370) for 11 obese men and from 2.2 to 12.6 (*P*<0.0001) for obese women. These increases in risk were large and particularly significant among women. However, among those with MetS, when the overweight and obese groups were compared with the normal-weight group, modest increases in the ORs were not statistically significant neither in men nor in women (*P*>0.2).

Life style modification intervention measures were not associated with 6-year risk of incident diabetes. When we repeated the analyses in participant not assigned to the lifestyle modification intervention the results remained essentially unchanged (Table 4). However, to capture full power (sample size) and information we did not split the original sample for final presentation.

**Table 4:** Contribution of combined effects of BMI and MetS to the risk of incident diabetes among individuals not assigned to lifestyle modification intervention measures

		Men		Women	
**MetS-BMI states**		**Odds ratio^a ^(95% CIs)**	**P value**	**Odds ratio^a ^(95% CIs)**	**P value**
**No MetS**	**BMI <25**	**1^b ^(reference group)**	**-**	**1^b ^(reference group)**	**-**
	BMI 25-30	1.3 (0.7-2.8)	0.415	1.9 (0.9-4.0)	0.115
	BMI ≥30	4.1 (1.5-11.0)	0.006	2.6 (1.1-6.1)	0.031
MetS	BMI <25	2.6 (1.0-6.7)	0.042	9.0 (3.1-26.0)	0.000
	BMI 25-30	2.5 (1.3-4.7)	0.007	6.4 (2.9-14.0)	0.000
	BMI ≥30	3.6 (1.8-7.3)	0.000	11.2 (5.4-22.9)	0.000

BMI, body a mass index, calculated as weight (kg) divided by height (m) squared; MetS, metabolic syndrome.

a. OR adjusted for age, family history of diabetes, history of CVD, education, and smoking.

b. Combination status of MetS-BMI was included as a polychotomous variable with 6 categories. Reference group was normal-weight individuals without MetS

## Discussion

For the first time, we studied the hypothesized sex-specific heterogeneity in the MetS status of men and women with normal weight, overweight, or obesity, with respect to the risk of incident diabetes. Consistent with previous studies, we demonstrated that BMI and MetS are significant predictors of diabetes [2,35,36]. This study further revealed that the magnitude of the association with MetS is greater than BMI, when examining the combined relationship of BMI and MetS. Joint analyses broaden our understanding of risk factors' relative influence on diabetes by showing that individuals with normal-weight/MetS phenotype had an increased risk of incident diabetes 3-9 times that of normal weight individuals without MetS; and that the overweight and obesity conferred an increased risk of incident diabetes both among those with and without MetS. Of importance was our finding that multivariate-adjusted risk of incident diabetes due to increased BMI began to appear earlier in women than in men.

There is no consensus regarding the definitions of body size phenotypes, the reported prevalence of the normal-weight/MetS and obese/without MetS phenotypes, thus, has been widely ranged 12 [15-18]. In this population-based study we observed that there were small numbers of men and women with normal weight who had MetS, resembling the normal-weight/MetS phenotype described by Ruderman and others [6-8] and modest numbers with obesity but without MetS, resembling the obese/without MetS phenotype described by Brochu, Karelis, and others [12-14,37]. The prevalence of the normal-weight/MetS and obese/without MetS phenotype in this study was 3.1% and 40.2%, respectively. The corresponding prevalence reported to be 8.6% and 65.8% among US adults [19], and 8.7% and 15.2% [18] among Korean adults.

The present findings indicate that normal-weight/MetS phenotype confers increased risk of incident diabetes, highlighting the importance of MetS among normal weight individuals. Contrariwise, overweight and obesity puts metabolically healthy individuals at not much so increased risk for developing diabetic. The magnitude of the association with diabetes risk for MetS was higher than that of obesity. These findings underscore the critical importance of MetS as a determinant of diabetes.

The presence of MetS augmented risk for incident diabetes, regardless of obesity status, therefore, screening in normal or slightly elevated BMI can help preventing diabetes [5]. Karelis et al [38] hypothesized and Meigs et al [10] showed that the normal-weight/MetS or obese/without MetS phenotypes exist in the community and have differential associations with diabetes. In line with previous studies[19], we observed that the normal-weight/MetS phenotype was associated with 3 and 9 fold risk factor adjusted OR for diabetes in men and women, respectively; accounting, respectively, for 5% to 6% of incident cases in this population. As expected, the highest diabetes incident rate (62%) was observed among overweight and obese individuals with MetS. Our results varied, however, from previous studies [19] in that we observed an increased risk of diabetes associated with obese/without MetS phenotype accounting for 22% of incident cases in this population.

Meigs et al cautioned the interpretation that individuals with obese/without MetS phenotype are really obese and healthy [10]. Data from Sweden refuted the notion that obese/without MetS phenotype is a benign condition [39]. In the interim, we could not confirm the existence of a healthy obese phenotype based on the absence of MetS. The TLGS participants with obese/without MetS phenotype were younger than their obese counterparts with MetS, it is still possible that the susceptible person has not yet developed the MetS; risk factor may begin to cluster in this subgroup as they age putting them at an increased risk for diabetes [10]. Trends in lipid profile have previously been documented to be more favorable among obese participants of the TLGS than among their overweight or normal weight counterparts [40]. The favorable trends in metabolic markers, however, may be annihilated by unfavorable trends in obesity [41]. This means that some of the overweight/obese participants without MetS at baseline might have developed MetS during follow-up, which in turn led to incident diabetes. This finding underscores the need for follow up for timely detection of deviation from normal metabolic state in this sub-groups [11]. More stringent definition rather than absence of MetS, need to be considered since the goal is to define true obese/without MetS population which is different from a non-MetS population [11,14].

The major strength of our sex-specific prospective study lies in the reliable follow up in a well-characterized population-based sample in which diabetes and its risk factors have been assessed with standardized measures both at baseline and follow up, systematically recording all of the variables required to the define MetS and completeness of ascertainment and accuracy of classification.

The interpretation of present data needs to be assessed within the context of the potential limitation of our study. First, the modest numbers of diabetic patients during 6.5 years follow up, might lead to inexact estimates for overweight men without MetS in sex stratified analysis adjusted for diabetic risk factors, no firm conclusions, therefore, should be drawn regarding this non significant association. Second, some misclassification of diabetes status may have occurred due to lacking confirmatory test for newly diagnosed diabetes. Third, there is an innate limitation to the concept of MetS, which has different definitions. We, however, have chosen among different definitions, the one that has been agreed upon by developers of different definitions of MetS [26]. Finally, it must be emphasized that the results of this study were determined in an Iranian urban adult population and further studies should be conducted to determine whether our findings are applicable to other populations.

## Conclusions

The results of this study establish that there is heterogeneity in BMI-metabolic risk sub-phenotypes in the population and that the MetS is a critical factor that confers risk for diabetes. Our finding highlights the importance of screening for MetS even in normal weight individuals, which is a laudable approach from a health education as well as a public health point of view and relates to the prevention of diabetes. In the presence of the MetS, increasing levels of BMI no longer contributed to the risk of incident diabetes. Furthermore, in participants without MetS, obesity increased risk for incident diabetes. Thus, in every day encounter as well as public health setting weight reduction measures, should be encouraged in conjunction with achieving metabolic targets not addressed by current definition of MetS.

## Competing interests

The authors declare that they have no competing interests.

## Authors' contributions

Conception and design: FH, MB. Analysis and interpretation of the data: FH, MS, MB. Drafting of the article: FH, MB. Critical revision of the article for important intellectual content: FH, MB, DK. Final approval of the article: FA, FH, MB, MS, DK. Statistical expertise: MS, MB. Collection and assembly of data: FA, MS, MB.

## Pre-publication history

The pre-publication history for this paper can be accessed here:

http://www.biomedcentral.com/1471-2458/11/383/prepub
